# Anterior wall myocardial infarction in a 16-year-old man caused by coronary artery aneurysm during the outbreak of COVID-19

**DOI:** 10.1186/s12872-020-01593-z

**Published:** 2020-07-01

**Authors:** Wenshuai Ma, Chunyu Li, Wei Zhang, Zhaole Ji, Yan Li

**Affiliations:** Department of Cardiology, Tangdu Hospital, Air Force Medical University, Xi’an, 710032 China

**Keywords:** Acute myocardial infarction, Coronary artery aneurysm, IVUS

## Abstract

**Background:**

Coronary artery aneurysm (CAA) is a potential cause of infarction. During the outbreak of coronavirus disease 2019 (COVID-19), home isolation and activity reduction can lead to hypercoagulability. Here, we report a case of sudden acute myocardial infarction caused by large CAA during the home isolation.

**Case presentation:**

During the outbreak of coronavirus disease 2019 (COVID-19),a 16-year-old man with no cardiac history was admitted to CCU of Tang du hospital because of severe chest pain for 8 h. The patient reached the hospital its own, his electrocardiogram showed typical features of anterior wall infarction, echocardiography was performed and revealed local anterior wall dysfunction, but left ventricle ejection fraction was normal, initial high-sensitivity troponin level was 7.51 ng/mL (<1.0 ng/mL). The patient received loading dose of aspirin and clopidogrel bisulfate and a total occlusion of the LAD was observed in the emergency coronary angiography (CAG). After repeated aspiration of the thrombus, TIMI blood flow reached level 3. Coronary artery aneurysm was visualized in the last angiography. No stent was implanted. Intravascular ultrasound (IVUS) was performed and the diagnosis of coronary artery aneurysm was further confirmed. The patient was discharged with a better health condition.

**Conclusions:**

Coronary artery aneurysm is a potential reason of infarction, CAG and IVUS are valuable tools in diagnosis in such cases, during the outbreak of coronavirus disease 2019 (COVID-19), home isolation and activity reduction can lead to hypercoagulability, and activities at home should be increased in the high-risk patients.

## Background

Coronary artery aneurysm (CAA) is a potential reason of infarction. During the outbreak of coronavirus disease 2019 (COVID-19), home isolation and activity reduction can lead to hypercoagulability. Here, we report a case of large CAA complicated with acute myocardial infarction (AMI) in a 16-year-old man during the home isolation.

## Case presentation

**Fig. 1:**
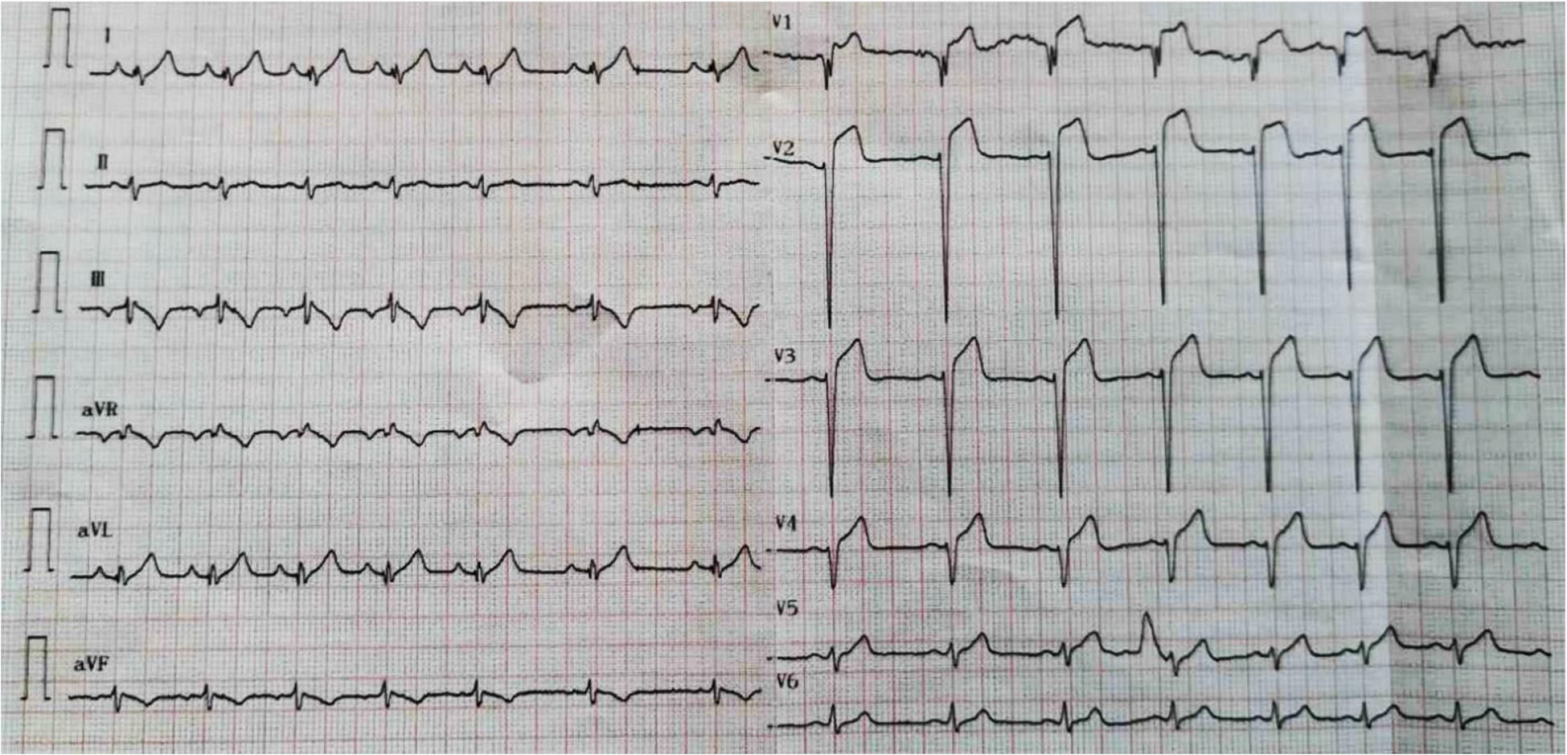
Electrocardiogram showing sinus tachycardia with ST-segment elevation on V1–5

**Fig. 2:**
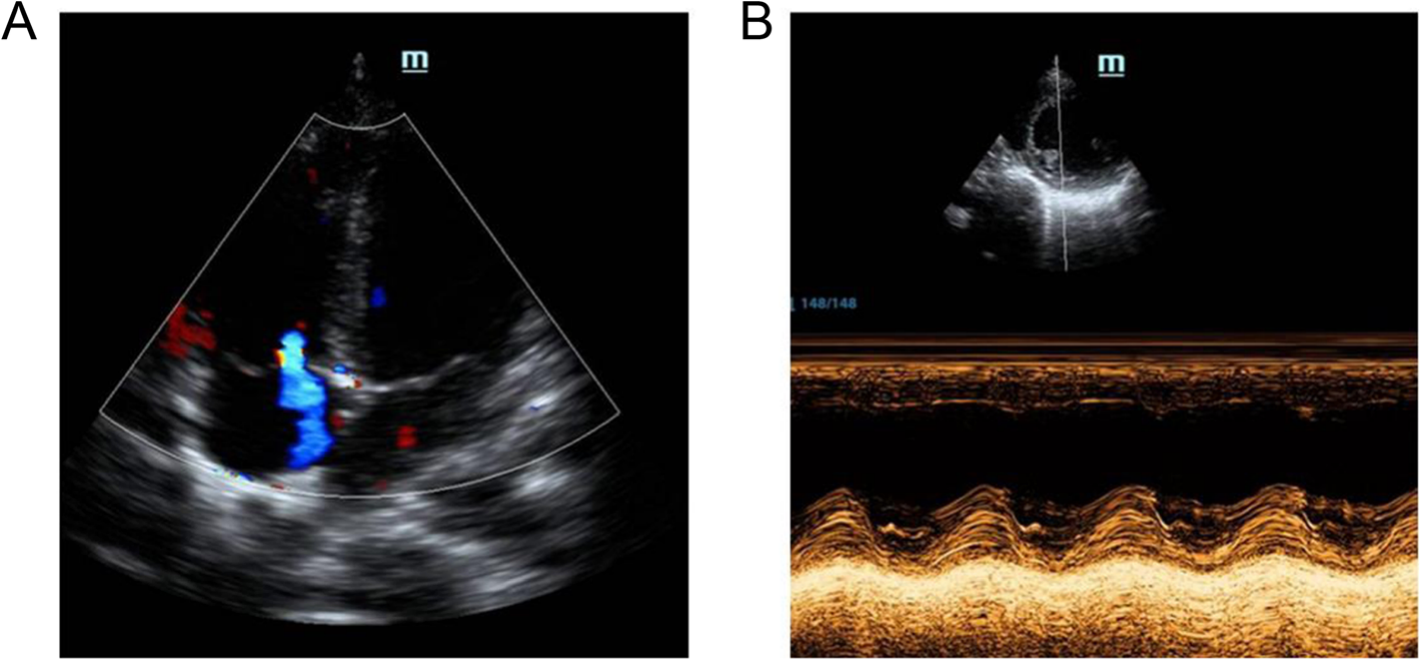
**a** In transthoracic echocardiography, local anterior wall dysfunction has been observed. **b** M-mode echocardiography showed left ventricle ejection fraction was good

**Fig. 3:**
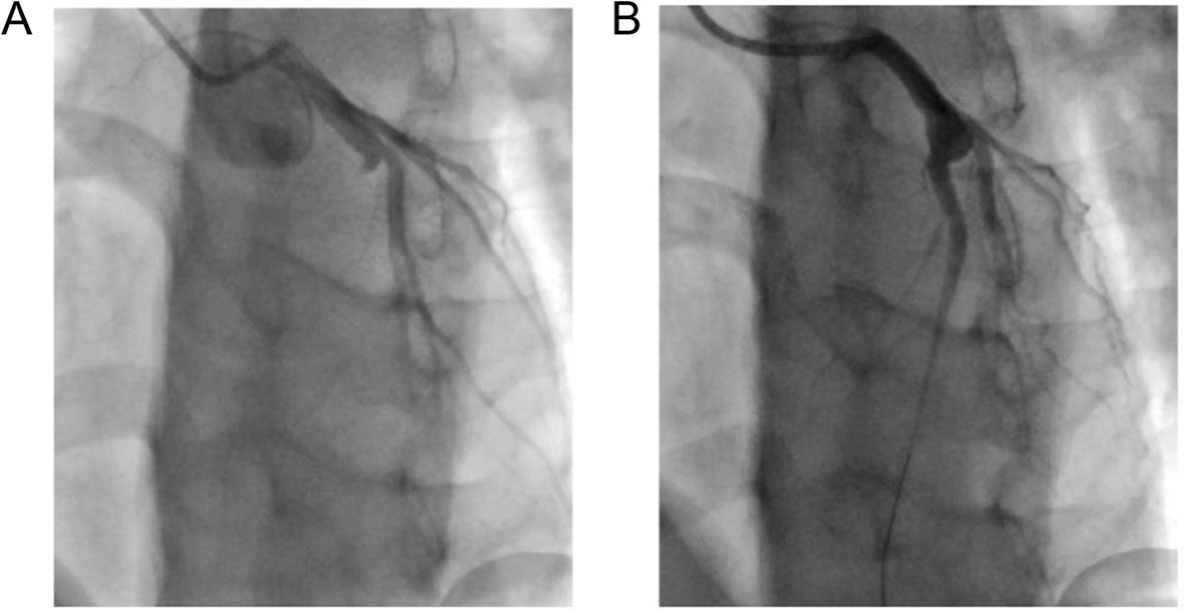
**a** Left coronary angiogram revealed a total occlusion of the LAD from the proximal segment; **b** Left coronary angiogram revealed a very large round aneurysm (arrowheads) originating from the proximal segment of the LAD

**Fig. 4:**
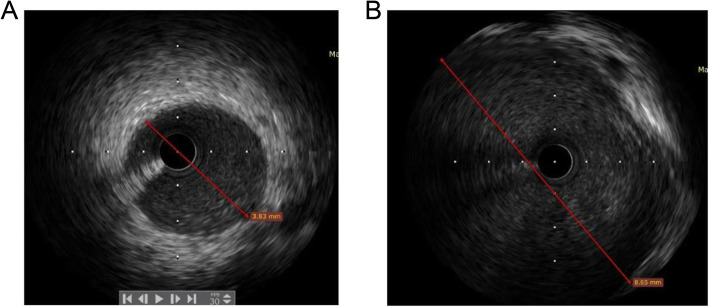
**a** IVUS showing normal LAD, the vessel diameter was 3.8 mm. **b** IVUS showing Coronary artery aneurysm, the widest segment was about 8.6 mm

**Fig. 5:**
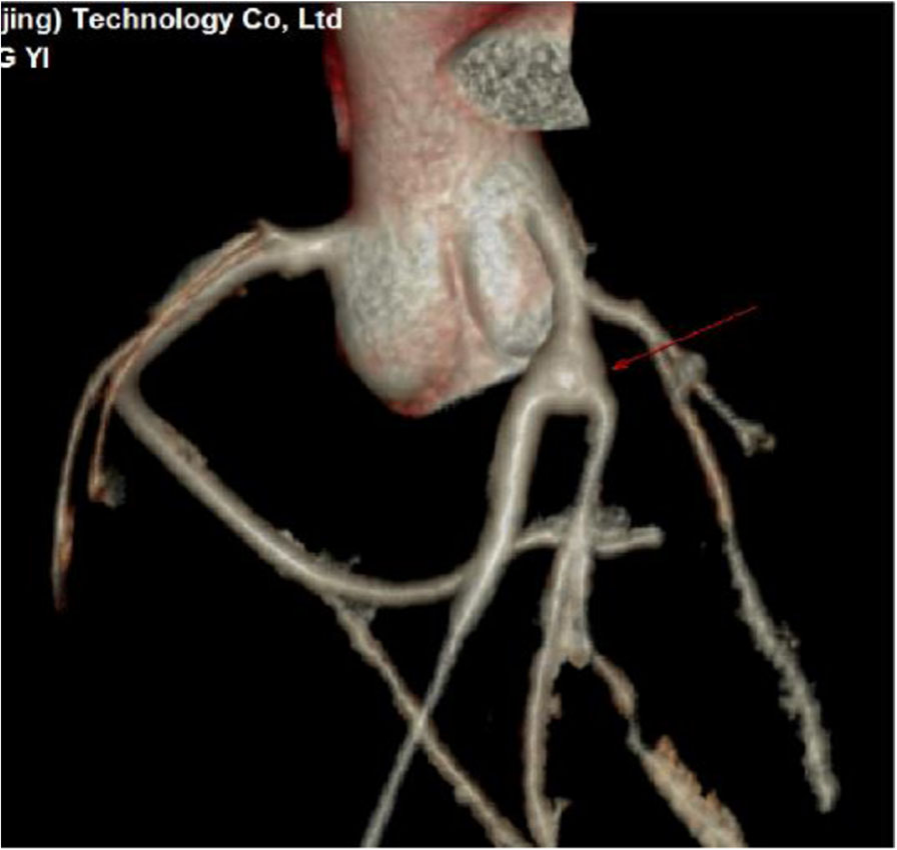
CTA showing a very large round aneurysm originating from the proximal segment of the LAD (red arrow indicating the aneurysm)

During the outbreak of coronavirus disease 2019 (COVID-19), a 16-year-old man with no cardiac history was admitted to CCU of Tang du hospital because of severe chest pain for 8 h. the patient reached the hospital its own. His cardiovascular examination revealed an initial blood pressure of 110/65 mmHg, heart rate of 95b.p.m.,body mass index (BMI)15.5 kg/m2, his electrocardiogram showed typical features of anterior wall infarction (Fig. 1) with a raised initial high-sensitivity troponin level which was 7.51 ng/mL (<1.0 ng/mL). On auscultation, his chest was clear and heart sounds were normal. In echocardiography, we found local anterior wall dysfunction, but left ventricle ejection fraction was normal (Fig. 2a, b). He had neither a history of hypertension, diabetes, smoking nor a family history of coronary heart disease. He had neither cold nor fever recently., and he denied the possibility of a past exposure to COVID-19. No medication was taken before admission. The patient received loading dose of aspirin and clopidogrel bisulfate, angiography that was performed immediately after transfer to the hospital, a total occlusion of the LAD from the proximal segment (Fig. 3a) was observed in the emergency coronary angiography (CAG). Right coronary artery and left circumflex artery were normal. A guidewire was successfully advanced across the occlusive lesion and a large fresh red thrombus was removed by aspiration catheter. After repeated aspiration of the thrombus and intra-coronary injection of tirofiban and urokinase, TIMI blood flow reached to level 3. Coronary artery aneurysm was visualized in the last angiography (Fig. 3b). Intravascular ultrasound (IVUS) was performed and further confirmed the diagnosis of coronary artery aneurysm (Fig. 4). No stent was implanted. ECG after the event showed resolution of MI pattern and evolution of infarction has been observed. After the emergency, results of laboratory assessments included normal levels of electrolytes, blood lipid and glucose, the C-reactive protein (CRP) level was 2.27 mg/L (0-3 mg/L) and erythrocyte sedimentation rate (ESR) was 20 mm/h (0–15 mm/h), NT-proBNP was 670 pg/ml, nucleic acid testing was negative, both inflammatory marker and rheumatoid factors were normal, ANA and other autoimmune markers were negative ruling out active connective tissue disease. The chest CT scan was normal. His test result for COVID-19 was negative. A computed tomography angiography (CTA) scan 5 days after admission showed that coronary artery aneurysm in the LAD (Fig. 5). the widest segment was about 8.6 mm. The patient was discharged home 7 d later on dual anti-platelet therapy (aspirin 100 mg and clopidogrel 75 mg).

## Discussion and conclusions

Coronary artery aneurysm is a potential reason of infarction. In older adults, Coronary artery aneurysm is associated with coronary atherosclerosis in more than 50% of cases. Other causes include Kawasaki’s disease (KD), Behcet’s Syndrome; Polyarteritis nodosa, Takayasu’s disease, Connective Tissue Disorders like Marfan syndrome, HIV, fungal, syphilitic Infections; use of illicit drugs like a mphetamine, cocaine [1] In our case, the patient is a 16-year-old man, in CAG, a total occlusion of the LAD was observed, but Right coronary artery and left circumflex artery were normal, and no atherosclerotic plaque was observed in IVUS. So the cause of infarction is not associated with atherosclerotic stenotic lesions. The patient and his parents denied the history of exposure to COVID-19, and his test result for COVID-19 was normal. They denied the history of Kawasaki’s disease, ANA and other autoimmune markers were negative ruling out active connective tissue disease, but maybe he had Kawasaki disease history when he was a child, but he and his parents did not know it.

The definition of coronary artery aneurysm is the internal diameter of coronary arteries dilatate > 1.5 times larger than the adjacent angiographically normal artery [2]. the diagnostic methods include CAG, IVUS and CTA. the CAG is an important diagnostic method for the coronary artery aneurysm. IVUS can not only accurately measure the diameter of aneurysm, but also distinguish between true aneurysm and pseudoaneurysm.

Coronary aneurysm was an obstructive ischemic coronary artery disease with impaired flow volume and may lead to exercise-induced myocardial ischemia [3]. The coronary flow reserve was also markedly reduced in the coronary aneurysms [4]. Computational fluid dynamics simulation has demonstrated that the hemodynamic parameters were different between coronary aneurysms and normal arteries specifically. The time-averaged wall shear stress (TAWSS) may be decreased and the oscillatory shear index (OSI) may be increased in aneurysms [5]. These changes could induce focal endothelial cell dysfunction and inflammation, which primes arterial regions for subsequent atherosclerosis initiation in response to hypercholesterolemia, and promote the transformation of stable plaque subtypes to unstable subtypes [6, 7]. And this hemodynamic changes in coronary aneurysms may increase blood viscosity and activates coagulation [8].

Coronary artery aneurysm is a potential cause of infarction. The treatments of CAA include medical, interventional and surgical therapy, because of there are no large clinical trials or guidelines in this subject., the treatment varies from doctor to doctor Medical treatments include antiplatelet agents, thrombolytic agents and anticoagulants [9]. In our case, we chose conservative management., dual anti-platelet therapy (aspirin 100 mg and clopidogrel 75 mg). The patient was in good condition during the 1-month follow-up. During the outbreak of COVID-19, home isolation and activity reduction can lead to hypercoagulability, infarction is more likely to happen., so activities at home should be increased in the high-risk patients.

## Data Availability

All relevant data is contained within the manuscript.

## Abbreviations

COVID-19: Coronavirus disease 2019
CCU: Cardiac care unit
IVUS: Intravascular ultrasound,
CAG: Coronary angiography
LAD: Left anterior descending
ECG: Echocardiography
MI: Myocardial infarction
BMI: Body mass index
CRP: C-reactive protein
PCI: Percutaneous coronary intervention
ESR: Erythrocyte sedimentation rate
ANA: Antinuclear antibodies
CTA: Computed tomography angiography
HIV: Human immunodeficiency virus
TAWSS: Time-averaged wall shear stress
OSI: Oscillatory shear index
